# Construction and analysis of degradome-dependent microRNA regulatory networks in soybean

**DOI:** 10.1186/s12864-019-5879-7

**Published:** 2019-06-28

**Authors:** Rui Wang, Zhongyi Yang, Yuhan Fei, Jiejie Feng, Hui Zhu, Fang Huang, Hongsheng Zhang, Ji Huang

**Affiliations:** State Key Laboratory of Crop Genetics and Germplasm Enhancement, College of Agriculture, Nanjing Agricultural University, Nanjing, 210095 China

**Keywords:** microRNA, Degradome, Regulatory network, Soybean, DDN

## Abstract

**Background:**

Usually the microRNA (miRNA)-mediated gene regulatory network (GRN) is constructed from the investigation of miRNA expression profiling and target predictions. However, the higher/lower expression level of miRNAs does not always indicate the higher/lower level of cleavages and such analysis, thus, sometimes ignores the crucial cleavage events. In the present work, the degradome sequencing data were employed to construct the complete miRNA-mediated gene regulatory network in soybean, unlike the traditional approach starting with small RNA sequencing data.

**Results:**

We constructed the root-, cotyledon-, leaf- and seed-specific miRNA regulatory networks with the degradome sequencing data and the forthcoming verification of miRNA profiling analysis. As a result, we identified 205 conserved miRNA-target interactions (MTIs) involved with 6 conserved gma-miRNA families and 365 tissue-specific MTIs containing 24 root-specific, 45 leaf-specific, 63 cotyledon-specific and 225 seed-specific MTIs. We found a total of 156 miRNAs in tissue-specific MTIs including 18 tissue-specific miRNAs, however, only 3 miRNAs have consistent tissue-specific expression. Our study showed the degradome-dependent miRNA regulatory networks (DDNs) in four soybean tissues and explored their conservations and specificities.

**Conclusions:**

The construction of DDNs may provide the complete miRNA-Target interactions in certain plant tissues, leading to the identification of the conserved and tissue-specific MTIs and sub-networks. Our work provides a basis for further investigation of the roles and mechanisms of miRNA-mediated regulation of tissue-specific growth and development in soybean.

**Electronic supplementary material:**

The online version of this article (10.1186/s12864-019-5879-7) contains supplementary material, which is available to authorized users.

## Background

MiRNAs, a class of ~ 21 nt non-coding endogenous small RNAs, play crucial roles in soybean growth, development and stress responses [1–6] by pairing to the target mRNAs to direct their post-transcriptional repression [7]. MiRNA itself does not have an open reading frame (ORF) or encode any protein, but it has a high degree of evolutionary conservatism among all kind of species and obvious different expression along with time and space transmission, showing its important statute in various biological processes of plants.

Since the first miRNA Line4 was discovered in larval development of nematode [8, 9], the discoveries of novel miRNAs were explosive with small RNA sequencing technique, providing influx of information for researchers. Then, the scholars started to explore the interactions and regulatory networks medicated by two or more miRNAs with the prosperity of next generation sequencing, all kinds of small RNA databases and bioinformatics tools, for instance, miRBase [10], psRNATarget service [11], bowtie [12] and Cytoscape 8 [13].

With such abundant small RNA datasets and tools, it is allowed to apply different patterns of biosynthesis into small RNA quantification and their expression detection. MiRNAs are generated from stem-loop pre-miRNAs while siRNAs are mainly exacted from double-stranded precursors. According to the latest criteria of miRNA annotation updated by Axetell and Meyers on *plant cell* [14], its length is limited to 20~24 nt, and mature 21-nt miRNA discomposes from double-strand RNA and form miRISC (miRNA-induced silencing complex) with Argonaute protein(AGO) family to repress translation of mRNAs or degrade mRNAs in the seed region [7, 15, 16]. The deep sequencing results of cleaved targets, generated latter, which is known as degradome, are used to validate the authenticity of the predicated MTIs.

Since the technique of degradome sequencing was performed to identify the miRNA-target relations in combination with rapid amplification of cDNA ends (RACE), high-throughput sequencing techniques and bioinformatics analysis [17], and mature protocols of Gene Ontology (GO) and Pathway analysis, we can understand thoroughly the regulation by miRNAs and it supports the results of bioinformatics and experimental supplement.

Simultaneously, some miRNAs have been reported with their specific regulation on different target genes in various biological pathways in plants. However, they tended to focus on miRNA expression performance and just with the predicted miRNA-Target relations, which usually results in incomplete regulatory relations. MiR172c was found to involve the repression of Auxin protein 2 (AP2) and to modulate the nod-related transcription factors NNC1 signaling pathway associated with nodule initiation in soybean [18]. Xu constructed miRNA-mediated gene regulatory networks in soybean cyst nematode (SCN) within 32 miRNA families and found six of them regulate the formation of SCN in root [19]. Even some genome-wide miRNA investigations, their explorations are also limited to expression verification. For instance, Chen did an investigation in soybean beginning with sRNA differential expression analysis under SMV infection, to find process-specific miRNAs, but most of the differentially expressed miRNAs did not have the high-efficiency cleavages based on degradome verification [20], which possibly dropped vital processes. The similar phenomenon showed up in Volkdin’s experiments about seed development [21].

MicroRNAs mediated gene regulatory networks generally started with investigating the miRNA’s expression profiling and target predictions so far. However, the level of miRNA’s expression cannot represent the level of cleavage, thus, such analysis focusing on expression level often ignores the crucial cleavage events corresponding with low-level expressed miRNAs. In the present work, reversely, the degradome sequencing data were employed to study the complete miRNA mediated regulatory networks in soybean. Based on degradome-dependent miRNA regulatory network (DDN) analysis, we found and validated conserved and specific MTIs, using tissue-specific MTIs to construct the root-, cotyledon-, leave- and seed-specific miRNA regulatory networks based on the degradome sequencing data, annotated with miRNA profiling analysis and discussed some co-interacted miRNA families mediated GRNs. All the results above indicate that the construction of DDN is a useful approach to describe a comprehensive regulatory network mediated by miRNAs, and will help botanist to learn further about the roles of miRNAs in soybean growth and development in the view of miRNA and target interactions.

## Results

### Construction and description of global DDNs

**Fig. 1:**
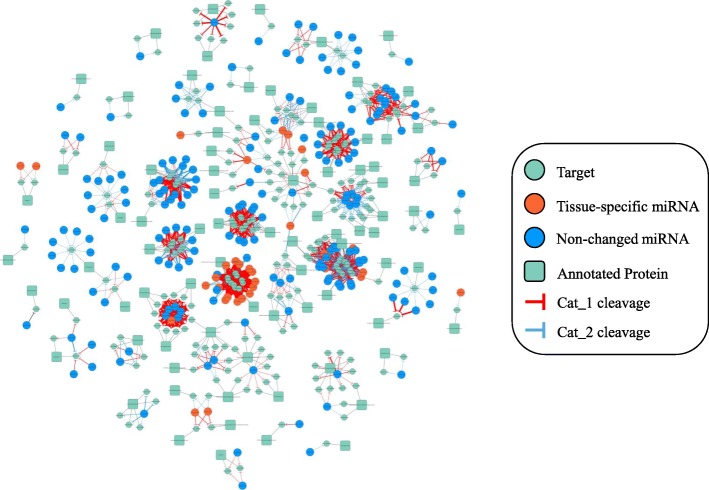
Degradome-dependent miRNA regulatory networks in soybean. The width of edges are gradient from 1 to 7, corresponding to the number of degradome libraries that verified the same MTIs. The color of edges represents the category of cleavage of highest degradome count (red is Cat_1 level, and blue is Cat_2).The grey-green circles represent targets, the grey-green squares represent target gene-encoding protein families (indicating biological function), blue circles are expression-non-changed miRNAs and orange ones are tissue-specific miRNAs

We took raw tables of degradome-dependent networks as fundamental information to classify the regulatory types of different miRNA mediated subnetworks, and studied further about conserved MTIs and tissue-specific MTIs. Based on the data predicted with psRNATarget server and relations validated by degradome data, we constructed a degradome-dependent miRNA regulatory network (Fig. 1). The grey-green circles represent target, the grey-green squares represent target gene-encoding protein family (indicating biological function), blue nodes are expression-non-changed miRNAs and orange ones are tissue-specific miRNAs. The width of edges is gradient from 1 to 7, representing the number of degradome libraries, which verified the same MTIs. The color of edges represents the category of cleavage of highest degradome count (red is Cat_1 level, and blue is Cat_2). It is obvious to observe that the combination of blue node and red edges accounting for large part of the verified networks. In the all-relation clusters, 9518 predicted relations were involved with 611 miRNAs, while 1804 of predicted MTIs were validated, involving 225 miRNAs.

**Fig. 2:**
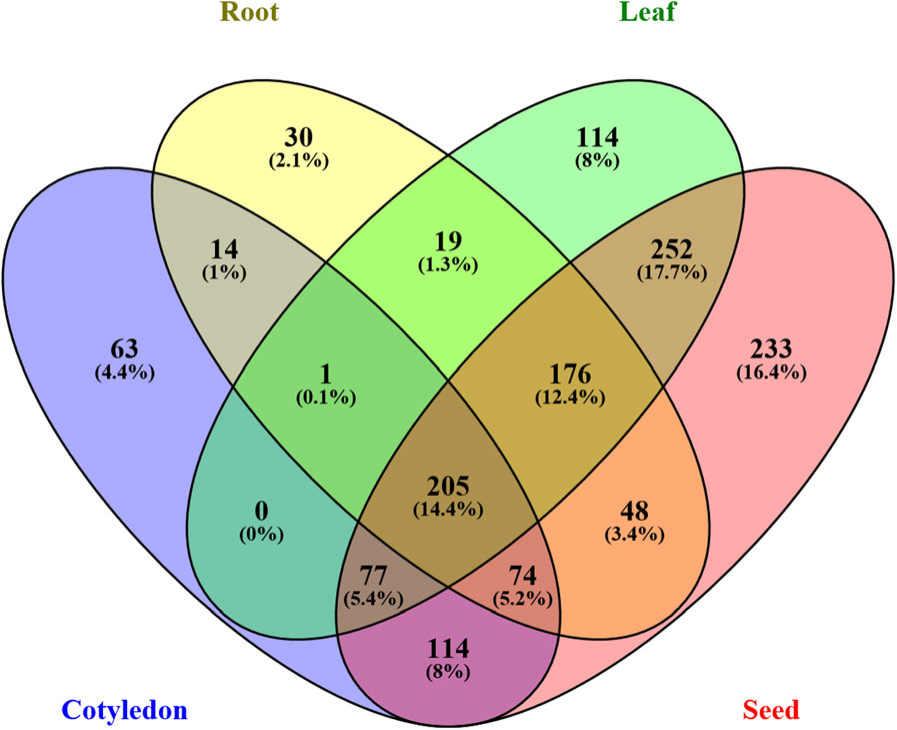
Venn diagram of diversity of gma-miRNA-Target relations in different tissues. The numbers in the picture show number of miRNA-Target relations’ distribution in root, leaf, cotyledon, and seed (including seed coat)

According to the distribution of gma-miRNAs from degradome-dependent networks, we found totally 205 tissue-conserved MTIs (Additional file 1: Table S1, Additional file 2: Table S2) and 440 tissue-specific MTIs (Additional file 3: Table S3, showing in form of a venn diagram Fig. 2), of which, 24 root-specific, 36 leaf-specific, 63 cotyledon-specific and 225 seed-specific MTIs were detected (Additional file 3: Table S3).

**Fig. 3:**
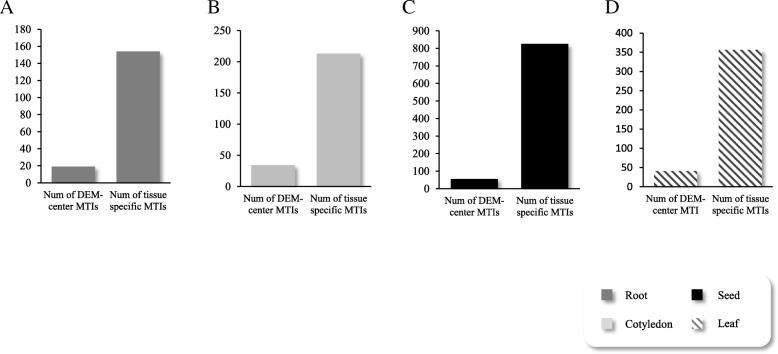
Comparisons of verified MTIs in different tissues. **a** Comparison of verified MTIs and DEM regulated MTIs in root; **b** Comparison of verified MTIs and DEM regulated MTIs in cotyledon; **c** Comparison of verified MTIs and DEM regulated MTIs in seed; **d** Comparison of verified MTIs and DEM regulated MTIs in leaf

Combining with identification of expression profile, we diagram occupation of differentially expressed miRNA (DEM) regulated MTIs in each tissue (Fig. 3): of 2561 MTIs verified in seed degradome, 60 are center with seed-specific DEMs; 13.6% MTIs (196 DEM-centered ones in all 1446 MTIs) verified by cotyledon degradome are DEM-center; 11.53% in leaf are mediated by leaf-specific DEMs, and none of the center miRNAs is root-specific in MTIs verified by root degradome. Difference between expression-non-changed miRNAs and DEM, distribution difference among different tissues can both indicate the different activity of miRNA in soybean. Detailed workflow is present in Additional file 5: Figure S1, and results of expression verifications were shown as a heatmap in Additional file 6: Figure S2.

### Identification of tissue-conserved sub-networks based on DDNs

**Fig. 4:**
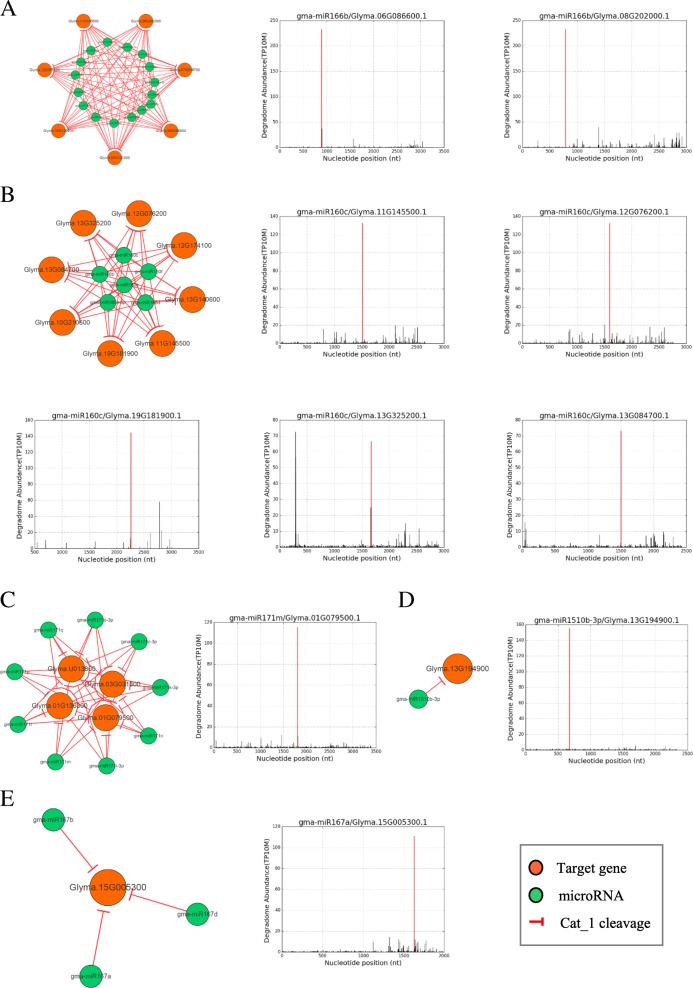
Tissue-conserved gma-miRNA family regulatory networks. **a** gma-miR166 family regulatory network; **b** gma-miR160 family regulatory network; **c** gma-miR171family regulatory network; **d** gma-miR1510 and **e** gma-miR167 regulatory networks

To identify the conserved cleavage events of miRNA-target other than the constitutively expressed miRNAs, we compared the DDNs constructed from various soybean tissues. As the MTIs of gma-miR166, gma-miR171, gma-miR160, gma-miR167, and gma-miR1510 families were found in all tissues’ DDNs and thus were considered as tissue-conserved sub-networks (Fig. 4). 16 gma-miR166 members have Cat_1-level cleavage on 7 targets, including homo zipper protein family gene and lipid-binding START domain-containing protein, 6 gma-miR160 members have the highest confident cleavage (Cat_1) on 7 targets, containing auxin response factor 10 (ARF 10), ARF 16 and ARF 17; 9 gma-miR171 members repressed 4 target genes, which are from GRAS family transcription factor, with Cat_1 cleavage; gma-miR167a/b/d have conserved MTIs with targets, encoding zinc finger (C3HC4-type RING finger) family protein; gma-miR1510-3p repressed gene encoding disease resistance proteins, the TIR-NBS-LRR class family proteins in legume [22] (Table 1).

**Table 1:** Gma-miRNA families from conserved MTIs

miR_family	Target_gene	Num_of_expressed_members	Num_of_degradome_libraries	Target_annotation
gma-miR1510	Glyma.13G194900	1	5	Disease resistance protein (TIR-NBS-LRR class) family
gma-miR160	Glyma.11G145500	6	7	auxin response factor 10
gma-miR160	Glyma.13G140600	6	7	auxin response factor 16
gma-miR160	Glyma.10G210600	6	7	auxin response factor 16
gma-miR160	Glyma.13G084700	6	7	auxin response factor 17
gma-miR160	Glyma.13G325200	6	6	auxin response factor 10
gma-miR160	Glyma.12G076200	6	7	auxin response factor 10
gma-miR160	Glyma.19G181900	6	6	auxin response factor 16
gma-miR160	Glyma.12G174100	6	6	auxin response factor 16
gma-miR166	Glyma.07G016700	16	6	lipid-binding START domain-containing protein
gma-miR166	Glyma.12G075800	16	6	lipid-binding START domain-containing protein
gma-miR166	Glyma.15G129700	16	6	lipid-binding START domain-containing protein
gma-miR166	Glyma.11G145800	16	6	lipid-binding START domain-containing protein
gma-miR166	Glyma.06G086600	16	6	homeobox gene 8
gma-miR166	Glyma.08G202000	16	6	lipid-binding START domain-containing protein
gma-miR166	Glyma.05G101500	16	6	homeobox gene 8
gma-miR167	Glyma.15G005300	3	7	zinc finger (C3HC4-type RING finger) family protein
gma-miR171	Glyma.U013800	9	6	GRAS family transcription factor
gma-miR171	Glyma.01G079500	9	6	GRAS family transcription factor
gma-miR171	Glyma.03G031800	9	6	GRAS family transcription factor
gma-miR171	Glyma.01G136300	9	6	GRAS family transcription factor

**Fig. 5:**
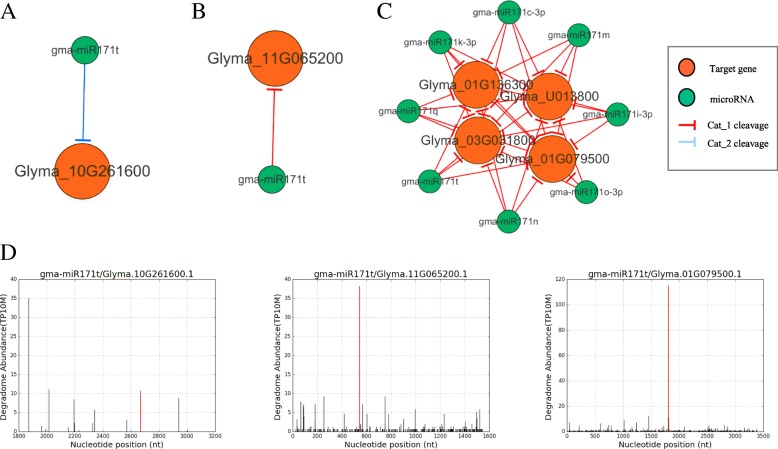
gma-miR171 mediated gene regulatory networks **a** Cotyledon-specific DDN **b** Seed-specific DDN **c** Conserved DDN; **d** Degradome verified cleavage t-plot of (**a**) (**b**) (**c**) *DDN, Degradome-Dependent MicroRNA-mediated networks. Red verticals in (**d**) are degradome count of the MTIs in figure titles, which show the cleavage level of MTIs

Some conserved miRNAs also had tissue-specific MTIs with different target genes. For example, tissue-conserved miRNA gma-miR171 had seed-specific MTIs regulating Glyma.11 g065200, Glyma.05g126100, and Glyma.08 g081100, specially regulated Glyma.10 g261600 in cotyledon and had specific target Glyma.01 g177200 in leaf, while in tissue-conserved network, repressed Glyma.01g079500, Glyma.01g136300, Glyma.03g031800, and Glyma.U013800 (Table 2, Fig. 5).

**Table 2:** Conserved gma-miR171 family regulatory networks

Category	miRNA	Target	Num_degradome	miRNA_seq	MTI-type	Target_annotation
Cat_2	gma-miR171t	Glyma.10G261600	1	TTGAGCCGCGTCAATATCTCA	Cotyledon-specific	Leucine-rich repeat protein kinase family protein
Cat_1	gma-miR171t	Glyma.11G065200	1	TTGAGCCGCGTCAATATCTCA	Seed-specific	GRAS family transcription factor
Cat_1	gma-miR171c-3p	Glyma.U013800	6	TTGAGCCGTGCCAATATCACA	Conserved	GRAS family transcription factor
Cat_1	gma-miR171c-3p	Glyma.01G079500	6	TTGAGCCGTGCCAATATCACA	Conserved	GRAS family transcription factor
Cat_1	gma-miR171c-3p	Glyma.03G031800	6	TTGAGCCGTGCCAATATCACA	Conserved	GRAS family transcription factor
Cat_1	gma-miR171c-3p	Glyma.01G136300	6	TTGAGCCGTGCCAATATCACA	Conserved	GRAS family transcription factor

Researchers may get the information of identical conserved MTIs above in Additional file 2: Table S2.

### Tissue-specific miRNA-mediated regulatory networks

Tissue-specific MTIs are particularly crucial to conclude the regulatory roles of these miRNAs in soybean. This approach only considers whether the MTIs are specific not the expression specificity of miRNAs. If degradome-specific MTI’s degradome read count is over 10 TP10M and miRNAs can be detected in certain tissue, we described them as tissue-specific MTIs. While those miRNAs whose expression in certain tissue is over twice of that in rest tissues (for each specific miRNA (SSR > Mean Square), we applied one-way ANOVA, when compared with the rest other tissues, if we observe log_2_(*Exp*_*specific*_/*Exp*_*other*_) > 2, then this miRNA is considered as tissue-specific miRNA), were considered as tissue-specific miRNAs. Among 365 degradome-specific MTIs, there are 24 root-specific, 45 leaf-specific, 63 cotyledon-specific and 225 seed-specific MTIs (Additional file 3: Table S3), however, in 22 miRNAs from cotyledon-specific MTIs, only miR156f is cotyledon-specific; miR4996 is the only leaf-specific one of all 33 miRNAs from leaf-specific MTIs and miR156b is the only seed-specific miRNA among 112 miRNAs from seed-specific MTIs. There is no consistently specific-expression miRNAs in root (Table 3). There are most abundant kinds of miRNAs from seed-specific MTIs, while distribution of miRNAs is similar in rest tissues.

**Table 3:** gma-miRNAs from tissue-specific MTIs

Tissue	Num	miRNA from tissue-specific MTIs
Cotyledon	22	gma-miR396k-5p,gma-miR159b-3p,gma-miR159c,gma-miR171i-3p,gma-miR171m, gma-miR396a-5p,gma-miR396e,gma-miR396c,gma-miR171q,gma-miR396b-5p,gma-miR396i-5p,gma-miR156f**,gma-miR159e-3p,gma-miR2118a-3p,gma-miR171o-3p,gma-miR171t,gma-miR171c-3p,gma-miR159f-3p,gma-miR2118b-3p,gma-miR159a-3p,gma-miR171k-3p,gma-miR168a
Leaf	33	gma-miR169d,gma-miR397b-5p,gma-miR1508c,gma-miR156d,gma-miR156e,gma-miR397a,gma-miR171d,gma-miR2109-5p,gma-miR169p,gma-miR156r,gma-miR171j-3p,gma-miR156m,gma-miR156i,gma-miR171f,gma-miR156l,gma-miR1510b-3p,gma-miR5674a,gma-miR156k,gma-miR156j,gma-miR5559,gma-miR1508a,gma-miR156t,gma-miR171g,gma-miR171e,gma-miR156o,gma-miR156f,gma-miR4996**,gma-miR5674b,gma-miR171u,gma-miR156n,gma-miR156c,gma-miR5672,gma-miR156p
Root	23	gma-miR4413b,gma-miR1515a,gma-miR399e,gma-miR399d,gma-miR1507c-3p,gma-miR1515b,gma-miR395a,gma-miR399b,gma-miR395m,gma-miR395k,gma-miR399g,gma-miR399h,gma-miR395l,gma-miR399a,gma-miR395b,gma-miR395j,gma-miR399f,gma-miR1510b-3p,gma-miR395c,gma-miR2109-5p,gma-miR395i,gma-miR399i,gma-miR399c
Seed	112	gma-miR164f,gma-miR159e-3p,gma-miR167f,gma-miR394b-5p,gma-miR167d,gma-miR396i-5p,gma-miR319g,gma-miR164k,gmamiR171i-3p,gma-miR156a,gma-miR164c,gma-miR159a-3p,gma-miR156w,gma-miR399e,gma-miR156x,gma-miR164a,gma-miR482c-3p,gma-miR164h,gma-miR5372,gma-miR171m,gma-miR164e,gma-miR319l,gma-miR394d,gma-miR530c,gma-miR394g,gma-miR171c-5p,gma-miR319f,gma-miR530e,gma-miR156u,gma-miR156s,gma-miR156y,gma-miR169n-3p,gma-miR164b,gma-miR164j,gma-miR156q,gma-miR5674b,gma-miR167c,gma-miR5674a,gma-miR396e,gma-miR171c-3p,gma-miR164g,gma-miR394c-5p,gma-miR164i,gma-miR396c,gma-miR530b,gma-miR395g,gma-miR396b-5p,gma-miR395f,gma-miR172d,gma-miR1510b-5p,gma-miR164d,gma-miR4393a,gma-miR172a,gma-miR394f,gma-miR394a-5p,gma-miR396a-5p,gma-miR171t,gma-miR159c,gma-miR159b-3p,gma-miR156h,gma-miR172b-3p,gma-miR530d,gma-miR156b**,gma-miR2119,gma-miR394e,gma-miR167e,gma-miR530a,gma-miR396k-5p,gma-miR156v,gma-miR1509b,gma-miR1507b,gma-miR167j,gma-miR1507c-3p,gma-miR167b,gma-miR1507a,gma-miR399d,gma-miR1515b,gma-miR2118a-3p,gma-miR394a-3p,gma-miR395e,gma-miR4996,gma-miR1509a,gma-miR171o-3p,gma-miR172l,gma-miR2109-5p,gma-miR171q,gma-miR9726,gma-miR319q,gma-miR159f-3p,gma-miR5785,gma-miR395d,gma-miR2118b-3p,gma-miR408d,gma-miR5772,gma-miR167a,gma-miR171k-3p,gma-miR172e,gma-miR172k,gma-miR399h,gma-miR9749,gma-miR399g,gma-miR167g,gma-miR399f,gma-miR1515a,gma-miR482a-3p,gma-miR399b,gma-miR172i-3p,gma-miR399c,gma-miR172h-3p,gma-miR172c,gma-miR399a,gma-miR172f

**, tissue-specific miRNAs. “Num” represents the number of miRNA from certain tissue. There are replicated detections of miRNAs among different tissues

**Fig. 6:**
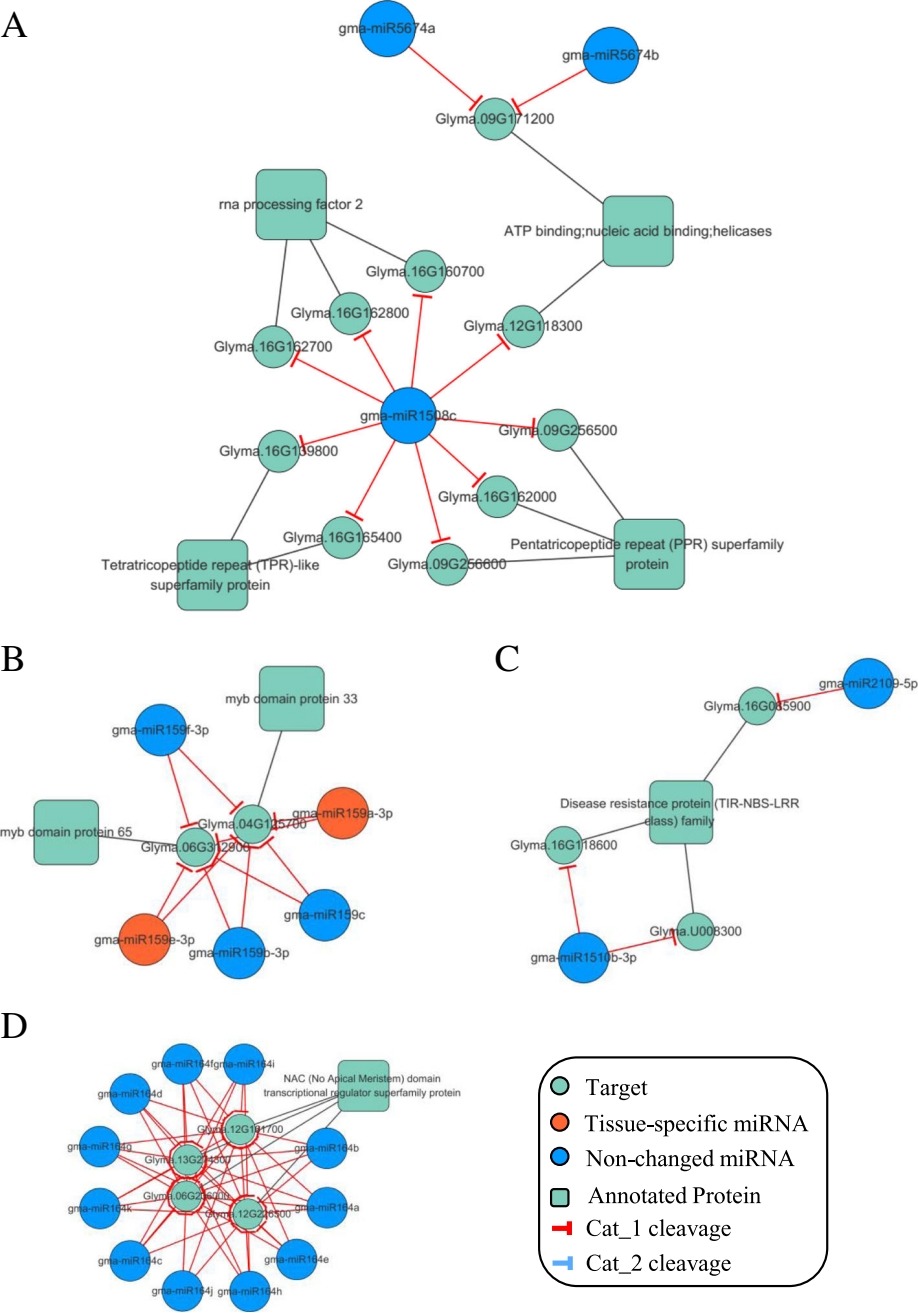
Exemplified tissue-specific subnetworks. **a** Leaf-specific DDN mediated by cotyledon-specific miRNA5674 and miRNA1508c **b** Cotyledon-specific miRNA159-mediated subnetwork **c** Root-specifc DDN regulated by miRNA1510b-3p and miRNA2109-5p; **d** Seed-specific miRNA164-mediated subnetworks. *DDN, Degradome-Dependent MicroRNA-mediated networks, ‘Tissues’ described whether the MTIs are conserved or tissue-specific.

We picked out tissue-specific subnetworks in every tissue--miR5674a and miR2109 co-mediated leaf-specific subnetworks, miR396 mediated cotyledon-specific subnetworks, miR164 mediated seed-specific subnetworks and root-specific networks, which were not tissue-specific miRNAs in certain tissue but had high efficiency of cleavage on targets (Fig. 6). Of these tissue-specific MTIs (listed in Additional file 3: Table S3), 45 are leaf-specific, 63 are cotyledon-specific, 24 are root-specific and 225 are seed-specific.

In leaf-specific networks, we found 45 leaf-specific MTIs, 29 of them were high-confident (Cat_1) cleavage and 16(the others) were less confident (Cat_2) MTI pairs. But in these networks, only 1 MTI is regulated by leaf-specific miRNA—gma-miR4996, having Cat_2 level cleavage on Glyma.15g070900. Except for the non-leaf-specific miR156 regulating SPL (Squamosa promoter-binding protein-like (SBP domain) transcription factor family protein) pairs, the root-specific miRNA gma-miR397a and gma-miR397b-5p were found specific targets Glyma.18g177400 and Glyma.08g359100, both encoding laccase 17 (Table 4). MiRNA5674 and miR1508c share the similar targets regulating ATP binding, nucleic acid binding helicases. Besides, miR1508c mainly regulates targets encoding Pentatricopeptide repeat (PPR) superfamily protein, Tetratricopeptide repeat (TPR)-like superfamily protein and rna processing factor 2 (Fig. 6a).

**Table 4:** Exemplified tissue-specific MTIs in different tissues

Category	miRNA	Target	miRNA_seq	Tissue	miRNA_type	Target_Annotation
Cat_1	gma-miR159b-3p	Glyma.04G125700	ATTGGAGTGAAGGGAGCTCCA	Cotyledon	non-changed	myb domain protein 33
Cat_1	gma-miR159e-3p	Glyma.06G312900	TTTGGATTGAAGGGAGCTCTA	Cotyledon	Leaf-specific	myb domain protein 65
Cat_1	gma-miR159e-3p	Glyma.04G125700	TTTGGATTGAAGGGAGCTCTA	Cotyledon	Leaf-specific	myb domain protein 33
Cat_1	gma-miR159a-3p	Glyma.06G312900	TTTGGATTGAAGGGAGCTCTA	Cotyledon	Leaf-specific	myb domain protein 65
Cat_1	gma-miR159c	Glyma.04G125700	ATTGGAGTGAAGGGAGCTCCG	Cotyledon	non-changed	myb domain protein 33
Cat_1	gma-miR159f-3p	Glyma.06G312900	ATTGGAGTGAAGGGAGCTCCA	Cotyledon	non-changed	myb domain protein 65
Cat_1	gma-miR1508c	Glyma.09G256500	TAGAAAGGGAAATAGCAGTTG	Leaf	non-changed	Pentatricopeptide repeat (PPR) superfamily protein
Cat_1	gma-miR1508c	Glyma.16G139800	TAGAAAGGGAAATAGCAGTTG	Leaf	non-changed	Tetratricopeptide repeat (TPR)-like superfamily protein
Cat_1	gma-miR1508c	Glyma.16G165400	TAGAAAGGGAAATAGCAGTTG	Leaf	non-changed	Tetratricopeptide repeat (TPR)-like superfamily protein
Cat_1	gma-miR1508c	Glyma.12G118300	TAGAAAGGGAAATAGCAGTTG	Leaf	non-changed	ATP binding;nucleic acid binding;helicases
Cat_1	gma-miR1508c	Glyma.16G162800	TAGAAAGGGAAATAGCAGTTG	Leaf	non-changed	rna processing factor 2
Cat_1	gma-miR1508c	Glyma.09G256600	TAGAAAGGGAAATAGCAGTTG	Leaf	non-changed	Pentatricopeptide repeat (PPR) superfamily protein
Cat_1	gma-miR5674b	Glyma.09G171200	TAATTGTGTTGTACATTATCA	Leaf	non-changed	ATP binding;nucleic acid binding;helicases
Cat_1	gma-miR1508c	Glyma.16G160700	TAGAAAGGGAAATAGCAGTTG	Leaf	non-changed	rna processing factor 2
Cat_1	gma-miR1508c	Glyma.16G162700	TAGAAAGGGAAATAGCAGTTG	Leaf	non-changed	rna processing factor 2
Cat_1	gma-miR1508c	Glyma.16G162000	TAGAAAGGGAAATAGCAGTTG	Leaf	non-changed	Pentatricopeptide repeat (PPR) superfamily protein
Cat_1	gma-miR5674a	Glyma.09G171200	TAATTGTGTTGTACATTATCA	Leaf	non-changed	ATP binding;nucleic acid binding;helicases
Cat_1	gma-miR2109-5p	Glyma.16G085900	TGCGAGTGTCTTCGCCTCTG	Root	non-changed	Disease resistance protein (TIR-NBS-LRR class) family
Cat_1	gma-miR1510b-3p	Glyma.U008300	TGTTGTTTTACCTATTCCACC	Root	non-changed	Disease resistance protein (TIR-NBS-LRR class) family
Cat_1	gma-miR1510b-3p	Glyma.16G118600	TGTTGTTTTACCTATTCCACC	Root	non-changed	disease resistance protein (TIR-NBS-LRR class), putative
Cat_1	gma-miR164e	Glyma.12G226500	TGGAGAAGCAGGGCACGTGCA	Seed	non-changed	NAC domain transcriptional regulator superfamily protein
Cat_1	gma-miR164e	Glyma.06G236000	TGGAGAAGCAGGGCACGTGCA	Seed	non-changed	NAC domain transcriptional regulator superfamily protein
Cat_1	gma-miR164a	Glyma.13G274300	TGGAGAAGCAGGGCACGTGCA	Seed	non-changed	NAC domain transcriptional regulator superfamily protein
Cat_1	gma-miR164c	Glyma.06G236000	TGGAGAAGCAGGGCACGTGC	Seed	non-changed	NAC domain transcriptional regulator superfamily protein
Cat_1	gma-miR164c	Glyma.12G161700	TGGAGAAGCAGGGCACGTGC	Seed	non-changed	NAC domain transcriptional regulator superfamily protein
Cat_1	gma-miR164k	Glyma.12G226500	TGGAGAAGCAGGGCACGTGCA	Seed	non-changed	NAC domain transcriptional regulator superfamily protein

Terms in ‘Tissue’ describe which tissue the MTI specifically occurs

In cotyledon-specific networks, there were 63 cotyledon-specific MTIs, one of them were mediated by tissue-specific miRNAs and showed a high-confidence cleavage. gma-miR159 regulated MYB domain protein genes; gma-miR168 repressed SSXT family protein genes; miR171 targeted on Leucine-rich repeat protein kinase family protein gene; gma-miR2118 regulated Tetratricopeptide repeat (TPR)-like superfamily protein and miR396 has regulation on growth-regulating factors. We take miR159-MYB subnetworks as examples of cotyledon-specific network, which is the center of both tissue-specific gma-miR159 (but not cotyledon-specific) and non-changed gma-miR159 (Fig. 6b). MiR159-MYB pattern was reported to induce by ABA and influenced seed germination in Arabidopsis in 2007 [23], the complex expression performance of repressor miR159 in rice indicates the multiple regulation between miR159 and MYB during seed germination.

In root-specific networks, there was no tissue-specific miRNA but 24 tissue-specific MTIs, including gma-miR1507c, 1515b, 2019-5p and 408c-3p, regulating mRNAs encoding LRR and NB-ARC domains-containing disease resistance proteins, dicer-like 2 [15], disease resistance protein (TIR-NBS-LRR class) family and plantacyanin. Some target genes are related to miRNA generation and regulation [7], like dicer-like 2. While these MTIs are mostly related to stress-response and primary biochemistry processes. We take miR2109 and miR1510-3p mediated subnetworks as example of root-specific networks (Fig. 6c).

In seed-specific networks, there are 225 seed-specific MTIs, including 3 seed-specific miRNA-center MTI—miR156b regulating Squamosa promoter-binding protein-like (SBP domain) transcription factor family protein (SPL), which is a typical miRNA-Transcription factor pattern. The rest seed-specific MTIs were mediated by non-specific miRNA but had high degraded read count, and of these MTIs, We found some published miRNA-target pairs, such as miRNA2119-ADH (alcohol dehydrogenase 1), a flooding-stress-resistant gene [24](alcohol dehydrogenase 1), miR164-NAC (No Apical Meristem domain transcriptional regulator superfamily protein), drought-response transcriptional regulator [25], and miR1515-dicer-like2 pairs. Seed-specific MTIs contain more abundant and high cleavage efficient regulatory relations between miRNA and target genes (Additional file 3: Table S3, Additional file 4: Table S4).

Detailed information of tissue-specific MTIs is present in Additional file 3: Table S3, miRNAs expression level in Additional file 4: Table S4.

### Interacted networks among different miRNA families in different tissues

**Fig. 7:**
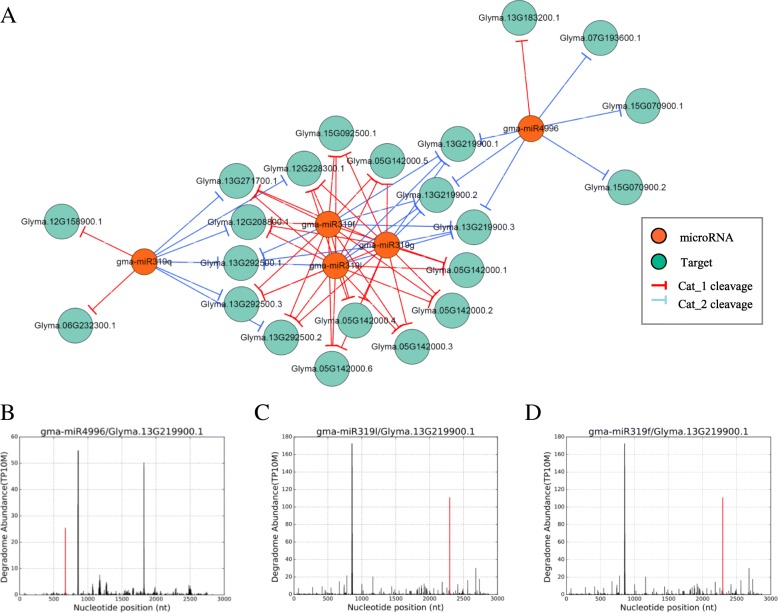
Interacted networks between different gma-miRNA families. **a** Co-interacted network of gma-miR 319 family and gma-miR4996, sub-network of Fig.3a; **b** Verified t-plot of gma-miR4996/Glyma.13 g219900, Y-aix represents degradome abundance(TP10M), X-aix represents nucleotide position (nt), the aligned region of microRNA on mRNA. **c** Verified t-plot of gma-miR319l/Glyma.13 g219900. **d** Verified t-plot of gma-miR319f/Glyma.13 g219900

By observing our constructed DDNs, we found an inter-families regulatory network involving miR319 family and miR4996 (Fig. 7), since our DDNs were mainly separated as every single miRNA-family regulatory group. They shared the target Glyma.13 g219900, which is homologous to TEOSINTE BRANCHED 1, cycloidea and PCF (TCP) transcription factor 2 in Arabidopsis, where miR319-regulated TCP has reported to control flowering time [26] . But their cleavage belongs to Cat_2 level, we hypothesized there might be a RNA edition of miRNA for higher probable cleavage on targets. So we found out the MTIs of miR319f/l and miR4996’s isoforms, which still showed no Cat_1 level cleavage on Glyma.13 g219900. It indicated that Glyma.13 g219900 probably is regulated by both miRNA and an uncharacterized small RNA.

### Difference analysis of networks regulated by the miRNAs from the same family

**Fig. 8:**
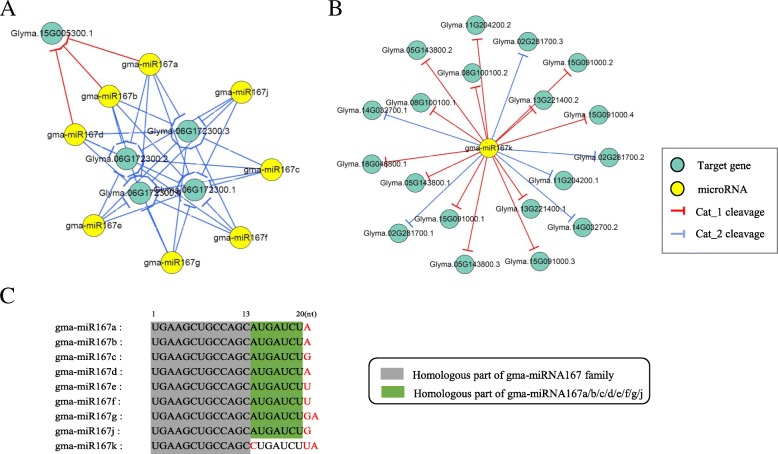
The gma-miR167 family regulatory network. **a** Gma-miRNA167 family regulatory networks (including miR 167a/b/c/d/e/f/g/j); **b** Gma-miRNA167k regulatory network; **c** Mature gma-miRNA167 sequence variation. The bases in red are sequence variations, the grey box is homologous part of gma-miRNA167 family, and the green box is homologous part of gma-miRNA167a/b/c/d/e/f/g/j

We observed that some miRNAs share totally different targets although they come from the same miRNA family. Different from the most miRNA family we detected, the members of miR167 family, as a typical example, do not work as a unit like others, but far apart into two general (Fig. 8), targets of gma-miR167k are mainly encoding auxin response factors, a regulator of plant growth and development from embryogenesis to senescence, GmARFs are reported to regulate the growth of soybean lateral root [27], other miRNA167 members repress zinc finger (C3HC4-type RING finger) family protein, nuclear protein X1 and HSP20-like chaperones superfamily protein, relating to abiotic stress response. To further dig out the possible molecular proof causing the huge functional difference, we compared the mature sequences of miRNA167 members and found that miR167k differentiate with other members in miR167 family from the 13rd base which is close to its seed region.

## Discussion

Usually the miRNA mediated GRN is constructed by investigating the miRNA expression profiling and target predictions. However, miRNA’s abundance sometimes cannot reflect whether these miRNAs, with high or low expression levels, play the crucial roles by cleaving the target genes in/under certain tissues or treatments. Specifically, the higher/lower level of miRNA does not mean the higher/lower level of cleavage. The same miRNAs may target different mRNAs in different tissues. Therefore, such analysis sometimes filters the crucial cleavage events where the corresponding miRNAs were lowly expressed. The degradome sequencing allows us to find out whether the cleavage events really occur and the cleavage abundances. Furthermore, whether the miRNA is expressed could be used to validate the degradome-dependent miRNA-target interaction in/under the certain tissues or treatments. This reverse approach may provide the reliable and complete miRNA-target interactions for network construction.

### Tissue-conserved MTIs and tissue-conserved miRNAs

By comparison of different networks constructed for different tissues/treatments, we can finally identify the crucial miRNAs and the miRNA-target interactions responsible for specific biological processes in plants. Besides getting rid of heavy laboratory work of identification, our method is more focused on the network level and discusses more about miRNAs’ cleavage efficiency in their MTIs .

Some conserved miRNAs we identified have been reported. Li’s team identified conservation and diversification of the miR166 family in soybean and discussed their potential roles [28], which we described as tissue-conserved miRNA family. Successively, some conserved MTIs were set as a solid model for further discussion. Turner reported miR160 regulated auxin responsive factor [29], Wang’s research discussed miR167-GmARF regulatory interactions’ function in soybean root development [27], and Xu found that miRNA167 in soybean nodules and lateral root growth can regulate auxin response factor, but we also detected gma-miR167 meditated MTIs showed up and high expressed in the soybean seed(Additional file 2: Table S2) and target on zinc finger (C3HC4-type RING finger) family protein, which was related to stress response in rice and hasn’t been reported in soybean yet.

However, tissue-conserved MTIs were not often regulated by conserved miRNA, and conserved miRNA also did not always in tissue-conserved MTIs. On the other side, their targets functioned in soybean development and disease resistance, indicating the conservatism of MTIs and miRNA families. Besides, some tissue-conserved miRNA families are identified to regulate totally different target genes in tissue-specific MTIs. For instance, miR171 is a conserved miRNA family in soybean, even miRNA171-GRAS regulatory pattern is conserved among different species, but in cotyledon, it has specific MTI with Leucine-rich repeat protein kinase family LRR-RPK) gene, a vital gene in regulation of leaf senescence.

Gma-miR171 in seed-specific MTIs regulated Glyma.11 g065200, Glyma.05 g126100, and Glyma.08 g081100, specially regulated Glyma.10 g261600 in cotyledon and had specific target Glyma.01g177200 in leaf, while in tissue-conserved network, repressed Glyma.01g079500, Glyma.01g136300, Glyma.03g031800, and Glyma.U013800 (Fig. 5). MiR171-GRAS pair is common and conserved in many other species, like *Gossypium hirsutum*, *Isatis indigotica* and so on [30–32]. But in soybean, besides GRAS, it also has cotyledon-specific MTIs with leucine-rich repeat protein kinase family protein gene.

### Tissue-specific MTIs and tissue-specific miRNAs

In this investigation, we found a total of 156 miRNAs in tissue-specific MTIs, however, only 3 miRNAs were detected consistently specific expression (Table 3). Of 365 tissue-specific MTIs, 40 are mediated by tissue-specific miRNA, while only 5 pairs are regulated by consistent specific miRNA, containing 3 seed-specific pairs, one leaf-specific pair and a cotyledon-specific one (seed-specific MTI is center with seed-specific miRNA) (Additional file 3: Table S3).

Tissue-specific miRNAs mediated MTIs we detected in this research only account for 8.8% of all tissue-specific MTIs. Tissue-specific MTIs required the high efficient cleavage (degradome count at least over 10 TP10M) of miRNA with different targets, so specific expression of miRNA is not necessary and tissue-specific miRNAs may have different tissue-specific MTIs for their different targets. MiR159e-3p, a cotyledon-specific miRNA and it targeted on MYB domain genes Glyma.04g125700 and Glyma.06g312900 with Cat_1 level cleavage, while in leaf it regulated Glyma.12g228100 and Glyma.13g271900, and had seed-specific MTIs in regulation of HCO3- transporter family genes Glyma.03g222300, Glyma.19g219500. Besides, these tissue-specific miRNAs may also get involved in conserved regulatory patterns, for example, leaf-specific miR156l regulated squamosa promoter binding protein-like 9 (SPL9) in both cotyledon and leaf, and in cotyledon miR156l detected higher cleaved efficiency (dependent on level of degraded target read count) than that in leaf, this pattern has reported in soybean to regulate the plant architecture [33]. Some tissue-specific miRNA may have different tissue-specific MTIs in other tissue, such as, leaf-specific miR1507c-3p has root-specific cleavage on Glyma.15g226100, encoding a LRR and NB-ARC domains-containing disease resistance protein.

On the other hand, those non-specific miRNAs can participate in some tissue-specific MTIs, related to some crucial biological and biochemistry processes because of their specific targets, for instance, gma-miR5674a, a non-changed miRNA, has a leaf-specific MTI with Glyma.09175800, homologous to NOP56-like pre RNA processing ribonucleoprotein in Arabidopsis. Of these MTIs, literally miRNA408c-3p-plantacyanin pair has been reported in Arabidopsis [34].

### Co-regulated networks by cross-family miRNAs and the specific networks regulated by the miRNAs from the same family

Overall our results, the distribution of 225 gma-miRNAs and their regulatory relations in different tissues are typically individuated into three conditions: (1) MiRNAs in soybean usually regulate as a unit of family, and their relations often perform as one-to-one, one-to-many or many-to-many form for regulating one or more target genes. The co-regulation between miRNAs often occurs in the same family. (2)Additionally, different miRNAs from different families have co-interaction with the unique genes, such as miR319f / g / l and miR4996 (Fig. 7). (3) Some miRNAs are isolated from other members in the same families, and can be used to correlate with multiple family members. It is independent of the main regulatory function group mediated by other family members, such as miRNA167k (Fig. 8) and other members in miR167 family (Fig. 8) and the causes for the differences may be the single base mutant in the conserved region or key sites on miRNA. According to the intersected networks, these universal MTIs validated in all the investigated tissues, and at least one member performed high expression in all tissues. These miRNA families are often considered as conserved miRNA families, often playing a key statue in the process of evolution and regulating the necessary biological processes, and generally have a co-cleavage of the same target transcript in several tissues, indicating that there are often over one members participating in the key metabolic processes to ensure the stability.

However, here is still boundedness of this method: 1) these validated MTIs are limited to the number of degradome libraries, and the expression verification still requires expression profiling data. 2) The expression of tissue-specific MTIs is hard to identify. The solution we adopted here is to combine small RNA expression data and degradome-verified cleavage data. With the increase of degradome sequencing data, it would be more convenient to study the miRNA-target interaction and build the miRNA mediated GRNs with the full branches of the interactions.

## Conclusions

The miRNA-target interactions (MTIs) and networks, not the miRNA themselves, should draw our attention when studying plant miRNA regulation. DDN approach may provide a more complete and reliable miRNA-target interactions, especially in the analysis of tissue/treatment-specific MTIs, and to study miRNA regulation in plants. Whether the abundances of MTIs can be directly predicted based on the abundance of the corresponding degradome reads is a question requiring further discussions and validations. In the present work, we constructed DDNs for four soybean tissues and identified the conserved and tissue-specific MTIs/sub-networks, which provides a basis of further studies of miRNA regulation in soybean growth and development.

## Methods

### Next generation sequencing (NGS) data

A total of 639 known miRNAs in soybean were extracted from miRbase [10](http://www.mirbase.org). The degradome libraries of different tissues (root, seed, cotyledon and leaf) were downloaded from NCBI GEO (Gene Expression Omnibus,http://www.ncbi.nlm.nig.gov/geo) under the accession numbers GSM1213430 [17], GSM647200 [35], GSM848963, GSM848964 [21] and GSM1419390, GSM1419391 [36](Table 5).

**Table 5:** Summary and description of employed datasets

Description	GEO_accession	Tissues	Developmental_stage/Treatment	Cultivar	Reference
sRNA-seq	GSM769282	Root	14 days after germination	Williams 82	Genes Dev 2011 Dec 1;25(23):2540–53.
sRNA-seq	GSM1419349	Leaf	water 30 min	Williams 82	Plant Cell 2014 Dec;26(12):4584–601.
sRNA-seq	GSM769285	Seed	15 days after flowering	Williams 82	Genes Dev 2011 Dec 1;25(23):2540–53.
sRNA-seq	GSM543394	immature seed coat	seeds of 50–75 mg fresh weight	Williams 82	Plant Cell 2009 Oct;21(10):3063–77
sRNA-seq	GSM543395	immature cotyledon	seeds of 50–75 mg fresh weight	Williams 82	Plant Cell 2009 Oct;21(10):3063–77
Degradome	GSM647200	Seed	15 days after flowering	Heinong44	BMC Plant Biol2011 Jan 10;11:5
Degradome	GSM825574	root,stem,leaf,inflorescence	four-week-old seedlings	Williams 82	Public on Nov 02, 2011
Degradome	GSM1213430	Root	2 days after transplant	Williams 82	Addo-Quaye et al.,2008
Degradome	GSM848963	Cotyledon	early maturation green 25–50 mg	Williams 82	BMC Genomics 2012 Jul 16;13:310.
Degradome	GSM848964	seed_coat	early maturation green 25–50 mg	Williams 82	BMC Genomics 2012 Jul 16;13:310.
Degradome	GSM1419390	Leaves	well-watered	IA3023	Plant Cell 2014 Dec;26(12):4584–601.
Degradome	GSM1419391	Leaves	Drought stressed	IA3023	Plant Cell 2014 Dec;26(12):4584–601.

### Processing of the degradome sequencing data

We employed the Cutadapt [37] to remove the adapters of the degradome sequencing reads, and then unify the forms of raw data sets. Normalization of the read count was Reads per 10 Million (RP10M), which was calculated by dividing the raw read counts with the total genome-mapped read counts and then multiplied by 10^7^. The processed degradome data are available in the DPMIND [38] repository (http://cbi.njau.edu.cn).

### MiRNA target prediction and degradome-based validation of miRNA-target pairs

We uploaded all the mature soybean miRNAs from miRBase v.22 into psRNATarget prediction server (http://plantgrn.noble.org/psRNATarget) [11] with default parameters and soybean cDNA library so that we could get the predicted results which describe the potential interactions between miRNAs and their target transcripts, and take these predicted results into the use of network construction for the observation of complicated regulatory interactions between miRNAs and target genes. First, the normalized degradome data should be mapped to the putative target sequences from prediction results; with the bowtie program, then the predicted miRNA-target pairs were retained if they met the following criteria: (1) there must be at least one degradome sequence with their 5’ends resided within 9~12 nt regions away from the 5’ends of the target binding sites; (2) read counts of at least one degradome sequence in above region should be more than 5RP10M; (3) read counts of one degradome sequence in above region should be the most abundant (Category 1) or higher than median (Category 2), among all the reads mapped to one target. Finally, the t-plot figures were generated, using our local developed python script.

### Classification of tissue-conserved and tissue-specific miRNA-target interactions (MTIs)

We imported degradome-verified results above into Cytoscape [13] (http://cytoscape.org) to construct raw regulatory networks of miRNA and their targets for different soybean tissues. To classify MTIs, we merged all seven tables of Degradome-dependent MiRNA Regulatory Networks (DDNs) in order to pick out overlapped MTIs in all four investigated tissues, which are treated as tissue-conserved MTIs, while those one-degradome-verified MTIs whose center miRNAs were detected express in certain tissue, are considered as tissue-specific MTIs. Simultaneously, we annotated the target genes with Phytozome.v11 Gmax_275_Wm82.a2.v1 (https://phytozome.jgi.doe.gov/pz/portal.html) [39] to prepare the discussion of DDN’s function (Additional file 5: Figure S1).

### Expression analysis of MiRNAs from degradome-classified MTIs

A home-made python script was in-house developed and was used to quantify miRNA expression in different small RNA sequencing libraries (libraries’ information are listed in Table 1). Then we merged all miRNA expression files in four tissues into an expression matrix and detected tissue-differential expressed miRNA with R package DESeq2 [40](Additional file 4: Table S4).

We applied one-way ANOVA to identify tissue-specifically expressed miRNAs:

For each specific miRNA (SSR > Mean Square), when compared with each rest tissue, if we can observe:

$$ {\log}_2\left({Exp}_{specific}/{Exp}_{other}\right)>2, $$

Then this miRNA is considered as tissue-specific miRNA. Of course, the miRNAs, failed to meet the criteria, are expression-non-changed miRNAs.

Conserved MTIs are those who can be verified in all tissues and center with miRNA expressing in over 2 tissues. Conserved miRNA families are those whose members are detected expressed in all different tissues.

## Additional files

Additional file 1: **Table S1.** Distribution of gma-miRNA-target interactions among different tissues. (XLSX 199 kb)

Additional file 2: **Table S2.** Basic information of conserved miR-target networks in investigated tissues (XLSX 46 kb)

Additional file 3: **Table S3.** Basic information of tissue-specific networks in identical tissue (XLSX 30 kb)

Additional file 4: **Table S4.** Expression verification of miRNAs in different tissues (XLSX 76 kb)

Additional file 5: **Figure S1.** Workflow of DDN construction and analysis. Summarize workflow of identifying tissue-specific and tissue-conserved MTIs and microRNAs in soybean in method with construction and analysis of DDNs. (PPTX 206 kb)

Additional file 6: **Figure S2.** Results of miRNA expression verification. A. heatmap of all DEMs B. qPCR results of miRNAs and targets from selected MTIs (PPTX 133 kb)

## Data Availability

The processed degradome data generated or analysed during the current study are available in the DPMIND repository http://cbi.njau.edu.cn, including GSM647200, GSM825574, GSM1213430, GSM848963, GSM848964, GSM1419390, GSM1419391, and small RNA seq data sets are available in Gene Expression Omnibus (GEO) repository https://www.ncbi.nlm.nih.gov/geo, including GSM769282, GSM1419349, GSM769285, GSM543394, GSM543395.

## Abbreviations

DDN: Degradome-dependent miRNA regulatory network
GRN: Gene Regulatory Network
MTI: MiRNA-Target Interaction

## References

[1] Ku YS, Wong JW, Mui Z, Liu X, Hui JH, Chan TF, Lam HM (2015). Small RNAs in plant responses to abiotic stresses: regulatory roles and study methods. Int J Mol Sci.

[2] Gupta M, Bhaskar PB, Sriram S, Wang PH (2017). Integration of omics approaches to understand oil/protein content during seed development in oilseed crops. Plant Cell Rep.

[3] Cao D, Li Y, Wang J, Nan H, Wang Y, Lu S, Jiang Q, Li X, Shi D, Fang C (2015). GmmiR156b overexpression delays flowering time in soybean. Plant Mol Biol.

[4] Xu S, Liu N, Mao W, Hu Q, Wang G, Gong Y (2016). Identification of chilling-responsive microRNAs and their targets in vegetable soybean (Glycine max L.). Sci Rep.

[5] Calvino M, Messing J (2013). Discovery of MicroRNA169 gene copies in genomes of flowering plants through positional information. Genome Biol Evol.

[6] Li XP, Gan R, Li PL, Ma YY, Zhang LW, Zhang R, Wang Y, Wang NN (2006). Identification and functional characterization of a leucine-rich repeat receptor-like kinase gene that is involved in regulation of soybean leaf senescence. Plant Mol Biol.

[7] Axtell MJ (2013). Classification and comparison of small RNAs from plants. Annu Rev Plant Biol.

[8] Reinhart BJ, Slack FJ, Basson M, Pasquinelli AE, Bettinger JC, Rougvie AE, Horvitz HR, Ruvkun G (2000). The 21-nucleotide let-7 RNA regulates developmental timing in Caenorhabditis elegans. Nature.

[9] Lee RC, Feinbaum RL, Ambros V (1993). The C. elegans Heterochronic gene lin-4 encodes small RNAs with antisense complementarity to lin-14. Cell.

[10] Kozomara A, Griffiths-Jones S (2011). miRBase: integrating microRNA annotation and deep-sequencing data. Nucleic Acids Res.

[11] Dai X, Zhuang Z, Zhao PX (2018). psRNATarget: a plant small RNA target analysis server (2017 release). Nucleic Acids Res.

[12] Langmead B, Trapnell C, Pop M, Salzberg SL (2009). Ultrafast and memory-efficient alignment of short DNA sequences to the human genome. Genome Biol.

[13] Su G, Morris JH, Demchak B, Bader GD (2014). Biological network exploration with Cytoscape 3. Curr Protoc Bioinformatics.

[14] Axtell MJ, Meyers BC (2018). Revisiting criteria for plant MicroRNA annotation in the era of big data. Plant Cell.

[15] Xie M, Zhang S, Yu B (2015). microRNA biogenesis, degradation and activity in plants. Cell Mol Life Sci.

[16] Reis RS (2017). The entangled history of animal and plant microRNAs. Funct Integr Genomics.

[17] German MA, Pillay M, Jeong DH, Hetawal A, Luo S, Janardhanan P, Kannan V, Rymarquis LA, Nobuta K, German R (2008). Global identification of microRNA-target RNA pairs by parallel analysis of RNA ends. Nat Biotechnol.

[18] Wang Y, Wang L, Zou Y, Chen L, Cai Z, Zhang S, Zhao F, Tian Y, Jiang Q, Ferguson BJ (2014). Soybean miR172c targets the repressive AP2 transcription factor NNC1 to activate ENOD40 expression and regulate nodule initiation. Plant Cell.

[19] Tian B, Wang S, Todd TC, Johnson CD, Tang G, Trick HN (2017). Genome-wide identification of soybean microRNA responsive to soybean cyst nematodes infection by deep sequencing. BMC Genomics.

[20] Chen H, Arsovski AA, Yu K, Wang A (2016). Genome-wide investigation using sRNA-Seq, Degradome-Seq and transcriptome-Seq reveals regulatory networks of microRNAs and their target genes in soybean during soybean mosaic virus infection. PLoS One.

[21] Shamimuzzaman M, Vodkin L (2012). Identification of soybean seed developmental stage-specific and tissue-specific miRNA targets by degradome sequencing. BMC Genomics.

[22] Yang S, Tang F, Gao M, Krishnan HB, Zhu H (2010). R gene-controlled host specificity in the legume-rhizobia symbiosis. Proc Natl Acad Sci U S A.

[23] Reyes JL, Chua NH (2007). ABA induction of miR159 controls transcript levels of two MYB factors during Arabidopsis seed germination. Plant J.

[24] Rizal G, Karki S. Alcohol dehydrogenase (ADH) activity in soybean (Glycine max [L.] Merr.)under flooding stress. Electron J Plant Breed. 2011;2(1):50–57.

[25] Hussain RM, Ali M, Feng X, Li X (2017). The essence of NAC gene family to the cultivation of drought-resistant soybean (Glycine max L. Merr.) cultivars. BMC Plant Biol.

[26] Liu J, Cheng X, Liu P, Li D, Chen T, Gu X, Sun J (2017). MicroRNA319-regulated TCPs interact with FBHs and PFT1 to activate CO transcription and control flowering time in Arabidopsis. PLoS Genet.

[27] Wang Y, Li K, Chen L, Zou Y, Liu H, Tian Y, Li D, Wang R, Zhao F, Ferguson BJ (2015). MicroRNA167-directed regulation of the auxin response factors GmARF8a and GmARF8b is required for soybean nodulation and lateral root development. Plant Physiol.

[28] Li X, Xie X, Li J, Cui Y, Hou Y, Zhai L, Wang X, Fu Y, Liu R, Bian S (2017). Conservation and diversification of the miR166 family in soybean and potential roles of newly identified miR166s. BMC Plant Biol.

[29] Turner M, Nizampatnam NR, Baron M, Coppin S, Damodaran S, Adhikari S, Arunachalam SP, Yu O, Subramanian S (2013). Ectopic expression of miR160 results in auxin hypersensitivity, cytokinin hyposensitivity, and inhibition of symbiotic nodule development in soybean. Plant Physiol.

[30] Zhang B, Liu J, Yang ZE, Chen EY, Zhang CJ, Zhang XY, Li FG (2018). Genome-wide analysis of GRAS transcription factor gene family in Gossypium hirsutum L. BMC Genomics.

[31] Heoa J-O, Changa KS, Kim IA, Lee MH, Leea SA, Songb S-K, Leeb MM, Lim J (2011). Funneling of gibberellin signaling by the GRAS transcription regulator SCARECROW-LIKE 3 in the Arabidopsis root. PNAS.

[32] Zhang L, Li Q, Chen J-F, Chen W-S (2016). Computational identification and systematic classification of novel GRAS genes in Isatis indigotica. Chin J Nat Med.

[33] Sun Zhengxi, Su Chao, Yun Jinxia, Jiang Qiong, Wang Lixiang, Wang Youning, Cao Dong, Zhao Fang, Zhao Qingsong, Zhang Mengchen, Zhou Bin, Zhang Lei, Kong Fanjiang, Liu Baohui, Tong Yiping, Li Xia (2018). Genetic improvement of the shoot architecture and yield in soya bean plants via the manipulation of GmmiR156b. Plant Biotechnology Journal.

[34] Song Z, Zhang L, Wang Y, Li H, Li S, Zhao H, Zhang H (2017). Constitutive expression of miR408 improves biomass and seed yield in Arabidopsis. Front Plant Sci.

[35] Song QX, Liu YF, Hu XY, Zhang WK, Ma B, Chen SY, Zhang JS (2011). Identification of miRNAs and their target genes in developing soybean seeds by deep sequencing. BMC Plant Biol.

[36] Arikit S, Xia R, Kakrana A, Huang K, Zhai J, Yan Z, Valdes-Lopez O, Prince S, Musket TA, Nguyen HT (2014). An atlas of soybean small RNAs identifies phased siRNAs from hundreds of coding genes. Plant Cell.

[37] Martin M (2011). Cutadapt removes adapter sequences from high-throughput sequencing reads. EMBnet J.

[38] Fei Y, Wang R, Li H, Liu S, Zhang H, Huang J (2018). DPMIND: degradome-based plant miRNA-target interaction and network database. Bioinformatics.

[39] Schmutz J, Cannon SB, Schlueter J, Ma J, Mitros T, Nelson W, Hyten DL, Song Q, Thelen JJ, Cheng J (2010). Genome sequence of the palaeopolyploid soybean. Nature.

[40] Love MI, Huber W, Anders S (2014). Moderated estimation of fold change and dispersion for RNA-seq data with DESeq2. Genome Biol.

