# Supplementary material for: Construction and analysis of degradome-dependent microRNA regulatory networks in soybean
Source: BMC Genomics. 2019 Jun 28;20:534. doi: 10.1186/s12864-019-5879-7 (PMC6599275; doi:10.1186/s12864-019-5879-7)
Supplement: Supplementary file 5 — Figure S1. Workflow of DDN construction and analysis. Summarize workflow of identifying tissue-specific and tissue-conserved MTIs and microRNAs in soybean in method with construction and analysis of DDNs. (PPTX 206 kb) [file 12864_2019_5879_MOESM5_ESM.pptx]

## Slide 1
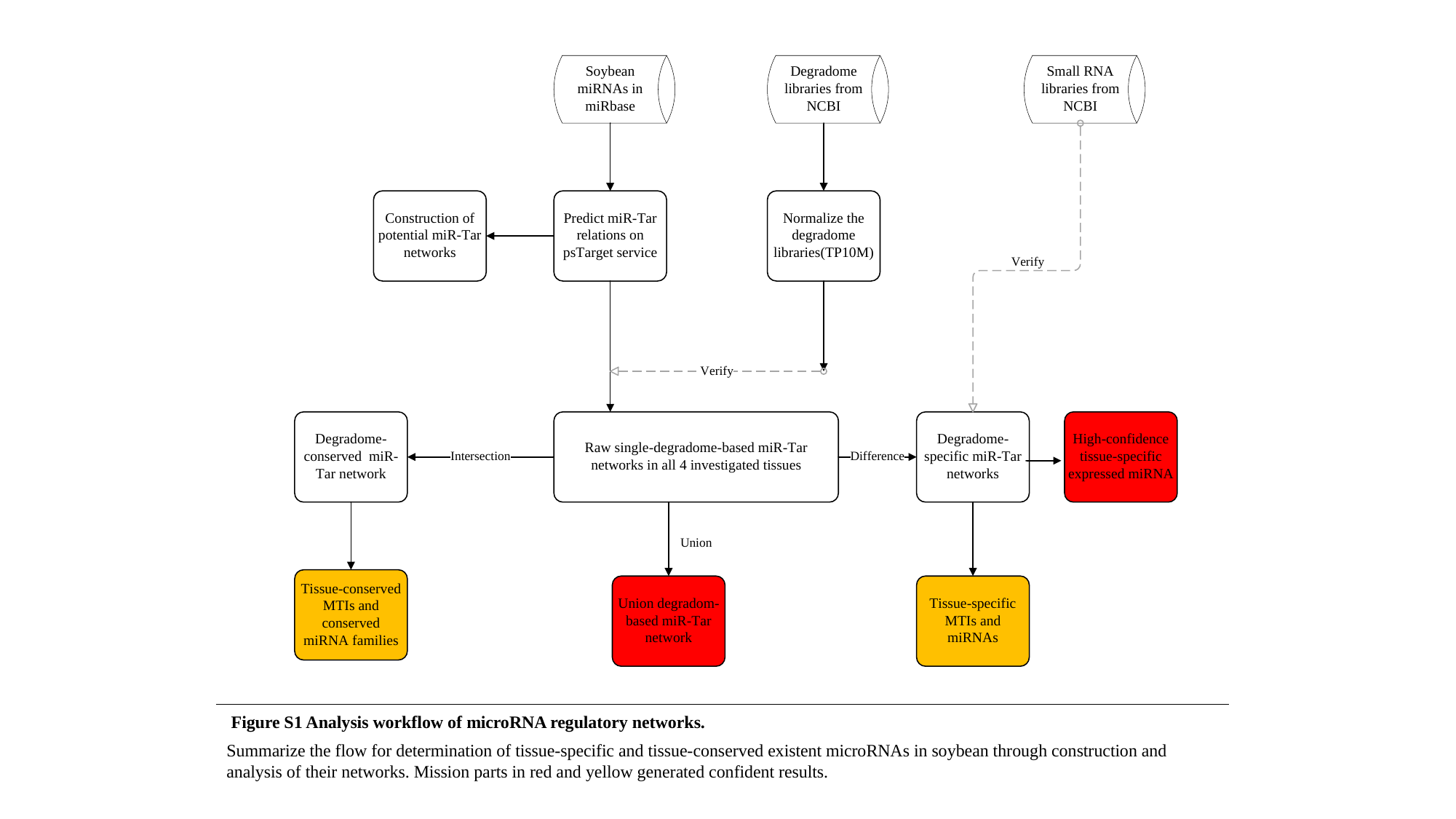

Figure S1 Analysis workflow of microRNA regulatory networks.
Summarize the flow for determination of tissue-specific and tissue-conserved existent microRNAs in soybean through construction and analysis of their networks. Mission parts in red and yellow generated confident results.
