# Supplementary material for: Construction and analysis of degradome-dependent microRNA regulatory networks in soybean
Source: BMC Genomics. 2019 Jun 28;20:534. doi: 10.1186/s12864-019-5879-7 (PMC6599275; doi:10.1186/s12864-019-5879-7)
Supplement: Supplementary file 6 — Figure S2. Results of miRNA expression verification. A. heatmap of all DEMs B. qPCR results of miRNAs and targets from selected MTIs (PPTX 133 kb) [file 12864_2019_5879_MOESM6_ESM.pptx]

## Slide 1
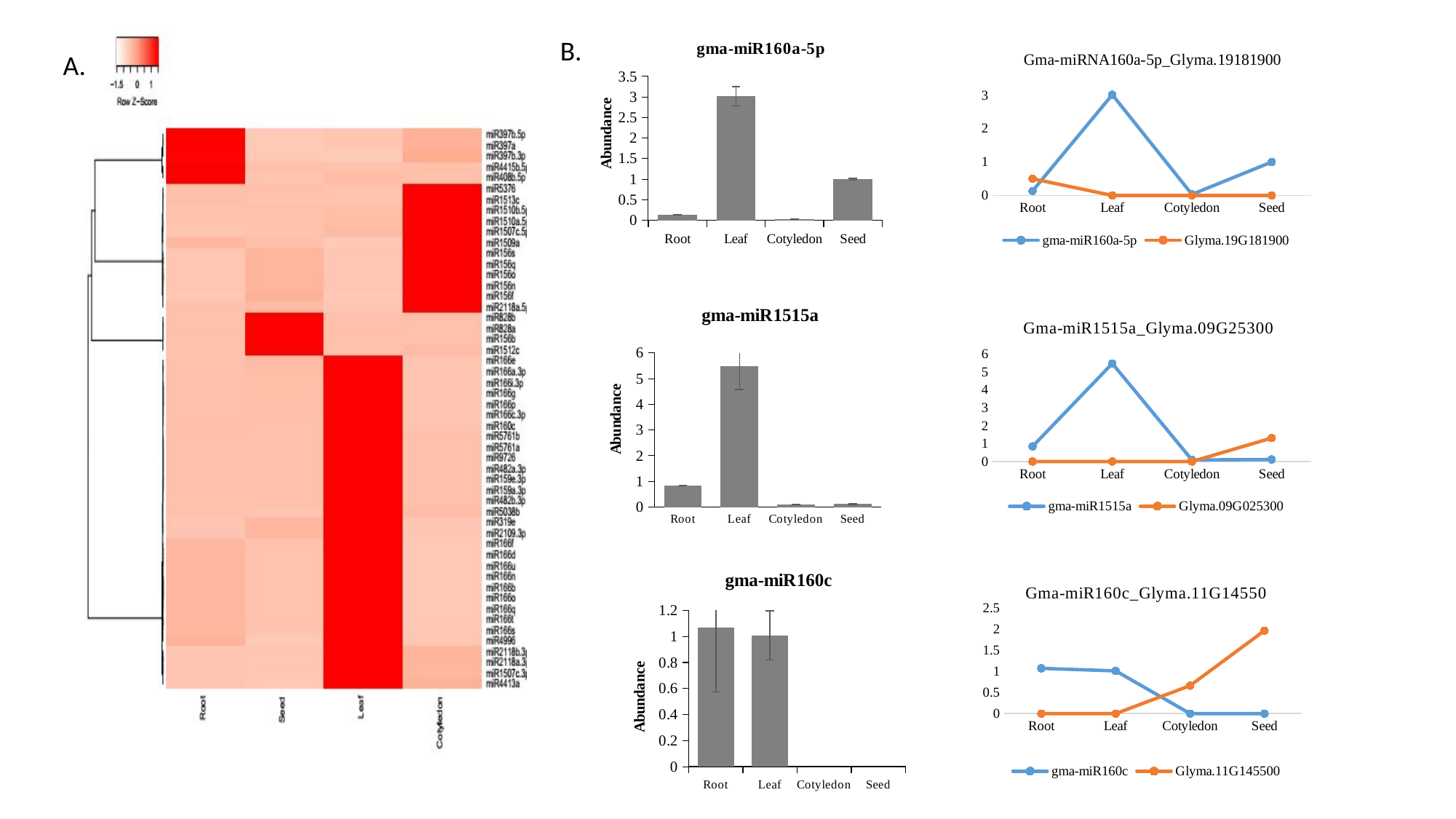

### Chart:
| Category | gma-miR160a-5p |
|---|---|
| Root | 0.135500514989951 |
| Leaf | 3.01623248777592 |
| Cotyledon | 0.0312434399882441 |
| Seed | 1.00016534116324 |
B.
### Chart: Gma-miRNA160a-5p_Glyma.19181900
| Category | gma-miR160a-5p | Glyma.19G181900 |
|---|---|---|
| Root | 0.135500514989951 | 0.498904932746301 |
| Leaf | 3.01623248777592 | 0.0 |
| Cotyledon | 0.0312434399882441 | 0.0 |
| Seed | 1.00016534116324 | 0.0 |A.
### Chart:
| Category | gma-miR1515a |
|---|---|
| Root | 0.845060123336459 |
| Leaf | 5.48216509546764 |
| Cotyledon | 0.0861216789399973 |
| Seed | 0.114950246122026 |
### Chart: Gma-miR1515a_Glyma.09G25300
| Category | gma-miR1515a | Glyma.09G025300 |
|---|---|---|
| Root | 0.845060123336459 | 0.0 |
| Leaf | 5.48216509546764 | 0.0 |
| Cotyledon | 0.0861216789399973 | 0.0 |
| Seed | 0.114950246122026 | 1.31080433234585 |
### Chart:
| Category | gma-miR160c |
|---|---|
| Root | 1.0681511405488 |
| Leaf | 1.00887093809339 |
| Cotyledon | 0.0 |
| Seed | 0.0 |
### Chart: Gma-miR160c_Glyma.11G14550
| Category | gma-miR160c | Glyma.11G145500 |
|---|---|---|
| Root | 1.0681511405488 | 0.0 |
| Leaf | 1.00887093809339 | 0.0 |
| Cotyledon | 0.0 | 0.659028860326339 |
| Seed | 0.0 | 1.95902166756264 |
